# Consumption of Energy Drinks among Italian University students: a cross-sectional multicenter study

**DOI:** 10.1007/s00394-023-03140-w

**Published:** 2023-04-06

**Authors:** Carmela Protano, Federica Valeriani, Andrea De Giorgi, Silvia Angelillo, Annalisa Bargellini, Aida Bianco, Lavinia Bianco, Giuseppina Caggiano, Maria Eugenia Colucci, Maria Anna Coniglio, Laura Dallolio, Osvalda De Giglio, Gabriella Di Giuseppe, Heba Safwat Mhmoued Abdo Elhadidy, Maria Eufemia Gioffrè, Pasqualina Laganà, Francesca Licata, Isabella Marchesi, Alice Masini, Maria Teresa Montagna, Christian Napoli, Giovanni Battista Orsi, Cesira Pasquarella, Concetta Paola Pelullo, Stefania Paduano, Rossella Sacchetti, Giacomo Scaioli, Roberta Siliquini, Licia Veronesi, Giorgio Liguori, Vincenzo Romano Spica, Matteo Vitali, Francesca Gallè

**Affiliations:** 1 Department of Public Health and Infectious Diseases, Sapienza University of Rome, Rome, Italy; 2 Department of Movement, Human, and Health Sciences, University of Rome “Foro Italico”, Rome, Italy; 3 Department of Health Sciences, School of Medicine, University of Catanzaro “Magna Græcia”, Catanzaro, Italy; 4 Department of Biomedical, Metabolic and Neural Sciences, Section of Public Health, University of Modena and Reggio Emilia, Modena, Italy; 5 Interdisciplinary Department of Medicine, University of Bari “Aldo Moro”, Bari, Italy; 6 Department of Medicine and Surgery, University of Parma, Parma, Italy; 7 Department of Medical and Surgical Sciences and Advanced Technologies “Gianfilippo Ingrassia”, University of Catania, Catania, Italy; 8 Department of Biomedical and Neuromotor Science, Alma Mater Studiorum University of Bologna, Bologna, Italy; 9 Department of Experimental Medicine, University of Campania “Luigi Vanvitelli”, Naples, Italy; 10 Department of Public Health Sciences and Pediatrics, University of Turin, Turin, Italy; 11 Multispecialty Clinical Institute, Trauma Orthopedic Care, Messina, Italy; 12 Department of Biomedical and Dental Sciences and Morphofunctional Imaging, University of Messina, Messina, Italy; 13 Department of Medical Surgical Sciences and Translational Medicine, Sapienza University of Rome, Rome, Italy; 14 Department of Movement Sciences and Wellbeing, University of Naples “Parthenope”, Via Medina 40, Naples, Italy; 15 Department of Education Studies “Giovanni Maria Bertin”, University of Bologna, Bologna, Italy; 16 City of Health and Science of Turin, Turin, Italy

**Keywords:** Energy drinks, Caffeine, University students, Unhealthy behaviors

## Abstract

**Purpose:**

The aim of the study was to evaluate the caffeinated Energy Drinks (EDs) consumption among a large sample of Italian undergraduates and its association with some of the major lifestyle risk factors.

**Methods:**

Students attending twelve public Italian universities were involved between October 2021 and May 2022. Information on socio-demographic characteristics, ED consumption, and on health-related behaviors of participants was collected by the use of a web-based questionnaire.

**Results:**

A total of 2165 students participated in the study and 15.2% of them reported having used caffeinated EDs in the last six months, mainly once a month (41.5%). In comparison with non-users, ED users showed a higher proportion of males (*p* < 0.001) and a higher father’s educational level (*p* = 0.003), came mainly from Northern universities (*p* = 0.004) and life sciences degree courses (*p* < 0.001). Besides, ED users reported higher BMI values (*p* = 0.003), more particular dietary regimens (*p* < 0.001), higher levels of weekly moderate–vigorous physical activity (*p* < 0.001) and participation in sports (*p* < 0.001) and in team sports (*p* = 0.003), and higher proportion of smokers (*p* < 0.001) and alcohol drinkers (*p* = 0.005). ED use was negatively related with female gender (OR 0.546; 95% CI 0.374–0.798), the Mediterranean diet (OR 0.587; 95% CI 0.362–0.951) and coming from the center of Italy (OR 0.500; 95% CI 0.275–0.909) and positively associated with tobacco smoke (OR 1.712; 95% CI 1.176–2.492) and participation in a team sport (OR 1.686; 95% CI 1.051–2.707).

**Conclusion:**

These findings could encourage figures engaged in education to increase the students’ awareness on this issue in order to prevent the excessive use of EDs and associated unhealthy behaviors, especially in the most interested subgroups.

## Introduction

Energy drinks (EDs) are defined as a beverage usually containing caffeine (also in large amounts), sugars, various additives, and different legal stimulants such as guarana, taurine, and l-carnitine [1]. EDs appeared for the first time in Europe and Asia in 1960 and exploded worldwide over the following years until to become a multibillion-dollar industry [2].

The popularity of EDs is due to the claim that these products provide an energy boost improving physical and cognitive performance [3].

Several studies in the field reported that EDs are consumed for compensating not enough sleep, boosting energy, increasing concentration during the study, driving for long periods, drinking with alcohol to improve the taste of drinks or treating hangovers [4]. For these reasons, the use of EDs is particularly popular among adolescents and young adults, especially university students. Several surveys [5–7] estimated the prevalence of EDs consumption in college students, reporting frequencies varying from 9 [5] to 90.6% [6]. This wide range is probably due to differences in data collection and study design.

These data cannot be neglected considering the great number of adverse effects associated to the consumption of EDs, such as increased heartbeat and blood pressure, arrhythmias, shakiness, dizziness, anxiety/nervousness/irritability, trouble sleeping, shaking and tremors, and stomachache, headache, heart palpitations, tingling/numbing skin, chest pain, dizziness, and addiction [8–13]. Several adverse effects determined by the use of EDs are associated with caffeine contained in these products. Indeed, the typical amount of added caffeine is equal to 7–32 mg/100 ml [14] and the European Food Safety Authority advises a safe level of 3 mg/kg bw per day of caffeine for children and adolescents [15]. High caffeine consumption may result in nausea, irritability, palpitations, and sleep disorders [16, 17].

Besides, the use of EDs seems to be associated also with other health-related behaviors such as alcohol use and/or smoke [18, 19] and scarce physical activity/sport [8, 20]. Thus, it is important to produce new data on the prevalence of EDs consumption and to study in depth the association between the use of EDs and unhealthy behaviors.

The aim of the study was to evaluate the caffeinated EDs’ consumption among a large sample of Italian university students and its association with some of the major lifestyle risk factors.

## Materials and methods

This study was nested in the “Study on Dietary Supplements Consumption”—DiSCo—cross-sectional study which was performed between October 2021 and May 2022 in twelve public Italian universities. A web-based questionnaire was used to collect information. Participants were asked to provide their informed consent before completing the questionnaire. The study was approved by the Research Committee of the University of Rome “Foro Italico” (approval n CAR 104/2021). All the procedures followed the principles of the Declaration of Helsinki.

### Participants

Students attending different degree courses at universities from northern, central, and southern Italy were invited to participate. Universities were selected by convenience. Since the estimated total undergraduate population included 468,967 individuals, a sample of at least 384 students would have been required assuming a 95% confidence level and a 50% response proportion.

### Questionnaire

A link to an anonymous questionnaire structured in a Google module was presented to the students by a researcher who described the aims of the study during a lecture. The questionnaire was built considering those used in previous investigations [21, 22]. The first section of the questionnaire was focused on socio-demographic information: gender, age, nationality, educational level of parents, university and degree course attended, and residential status (resident in the university area/commuter/non-resident but domiciled in the university area). The second section was aimed at exploring the health-related behaviors of participants: weight and height to obtain their BMI, and information about the type of dietary regimen adopted (no particular regimen/Mediterranean diet/other regimens), tobacco (smoker/quitter/no smoker) and alcohol use (yes/no, during meals or not, frequency of use), weekly time spent in moderate–vigorous activities (MVPA), practice of sport (yes/no) and its features (amateur/competitive level; endurance/strength sport; individual/team sport). The third section was used to specifically investigate the undergraduates’ habits of EDs consumption. In particular, students were asked to refer about their use of caffeinated energy supplements in the previous six months. Consumers were also asked to report the reason for their consumption (my own knowledge/physician or nutritionist’s indication/trainer’s suggestion/relative, friend, teammate’s suggestion/advertising), the aim pursued (to supplement the diet with caffeine/other ingredients, improve general health, improve appearance, recover after an effort, improve physical performance, improve mental performance, taste appreciation), the purchasing channel (shop or supermarket/specialized website/general trade website/sports facility) and the possible adverse events suffered in relation to EDs use.

### Statistical analyses

A descriptive analysis was performed on the participants’ characteristics, grouped by the use of EDs. Due to their non-normal distribution, continuous variables were expressed as median and Inter-Quartile Range (IQR), while categorical variables were summarized as the number and percentage of respondents for each group. The Mann–Whitney test and the chi-squared test were used to compare continuous and categorical variables, respectively, between users and non-users. All those variables which showed significant differences in the two groups were included in a logistic regression analysis. For these purposes, the use of EDs in the previous 6 months was considered as an outcome (expressed as no use = 0, use = 1) and the independent variables were categorized as follows: female gender = 0, male = 1; age < median value = 0, ≥ median value = 1; university from North Italy = 0, Center = 1, South = 2; life science area of study = 0, other = 1; mother’s or father’s educational mandatory level = 0, high school = 1, degree or more = 2; underweight = 0, normal weight = 1, overweight = 2, obese = 3; no particular diet = 0, Mediterranean diet = 1, other regimens = 2; moderate-to-vigorous physical activity (MVPA) level < median value = 0, ≥ median value = 1; no sports practice = 0, sports practice = 1; endurance sport = 0, strength sport = 1; individual sport = 0, team sport = 1; no smoker = 0, smoker = 1, quitter = 2; no alcohol use = 0, alcohol use = 1.

Statistical analyses were conducted using the Statistical Package for Social Science (SPSS) version 28.0 (IMB; Armonk, NY, USA).

## Results

A total of 2,165 completed the online questionnaire. Three-hundred and twenty-nine of them (15.2%) reported the use of caffeinated EDs in the last six months.

Table 1 shows the results of the comparison performed on sociodemographic and behavioral characteristics of users and non-users.

**Table 1:** Characteristics of participants grouped by ED consumption with related p value

Variable	ED users *n* = 329	ED non-users *n* = 1836	*p* value
Age			
Median (IQR)	22 (20–24)	22 (20–23)	0.854^a^
Gender (*n*, %)			
Female	173 (52.6)	1333 (72.6)	< 0.001^b^
Male	156 (47.4)	503 (27.4)	
Geographical area (*n*, %)			
North	185 (56.2)	850 (46.3)	0.004^b^
Center	36 (10.9)	233 (12.7)	
South	108 (32.8)	753 (41.0)	
Study area (*n*, %)			
Life sciences	253 (76.9)	1371 (74.7)	< 0.001^b^
Other	76 (23.1)	464 (25.3)	
Residential status			
Resident in the university area	112 (34.0)	592 (32.2)	0.621^b^
Commuter	97 (29.5)	590 (32.1)	
Domiciled in the area	120 (36.5)	654 (35.6)	
Mother’s educational level			
Mandatory	63 (19.1)	394 (21.5)	0.583^b^
High school	166 (50.5)	880 (47.9)	
Degree	100 (30.4)	562 (30.6)	
Father’s educational level			
Mandatory	75 (22.8)	487 (26.5)	0.003^b^
High school	135 (41.0)	854 (46.5)	
Degree	119 (36.2)	495 (27.0)	
BMI			
Median (IQR)	22.1 (20.3–24.6)	21.5 (19.8–23.9)	0.003^a^
Underweight	23 (7.0)	156 (8.5)	< 0.001^b^
Normal weight	236 (71.7)	1371 (74.7)	
Overweight	65 (19.8)	233 (12.7)	
Obese	5 (1.5)	76 (4.1)	
Diet			
No particular regimen	136 (41.3)	741 (40.4)	< 0.001^b^
Mediterranean diet	63 (19.1)	553 (30.1)	
Weight-loss diet	4 (1.2)	185 (10.1)	
Vegetarian/vegan diet	37 (11.2)	87 (4.7)	
High-protein diet	7 (2.1)	87 (4.7)	
Low-carbohydrate diet	36 (10.9)	27 (1.5)	
Weight-increase diet	20 (6.1)	32 (1.7)	
Low-fat diet	5 (1.5)	66 (3.6)	
High-carbohydrate diet	20 (6.1)	29 (1.9)	
Low-sodium diet	1 (0.3)	4 (0.2)	
MVPA/week (min)			
Median (IQR)	180 (40–300)	120 (30–240)	< 0.001^a^
Sport			
No	123 (37.4)	910 (49.6)	< 0.001^b^
Amateur	170 (51.7)	767 (41.8)	
Competitive	36 (10.9)	159 (8.7)	
Endurance	102 (49.5)	495 (53.5)	0.305^b^
Strength	104 (50.5)	431 (46.5)	
Individual	153 (74.3)	771 (83.3)	0.003^b^
Team	53 (25.7)	155 (16.7)	
Smoke habit (*n*, %)			
No smokers	168 (51.1)	1200 (65.4)	< 0.001^b^
Smokers	143 (43.5)	553 (30.1)	
Quitters	18 (5.5)	83 (4.5)	
Alcohol use			
No	30 (9.8)	277 (16.0)	0.005^b^
Yes	277 (90.2)	1456 (84)	
During meals	111 (41.3)	623 (42.5)	0.660^b^
Between meals	158 (58.7)	842 (57.5)	
≤1 times/month	66 (23.8)	421 (28.9)	
2–4 times/month	138 (49.8)	770 (52.9)	0.003^b^
2–3 times/week	61 (22.0)	237 (16.3)	
≥ 4 times/week	3 (1.0)	6 (0.4)	
Every day	9 (3.2)	22 (1.5)	

^a^Mann–Whitney test

^b^Chi-squared test

Users showed an almost equal gender distribution, attended mainly universities from North Italy and degree courses in the life sciences area, were mostly resident or domiciled in the university area, and reported most frequently a high school educational level for both parents. The majority of them reported normal weight, no particular dietary regimens, a median amount of 3 h/week of MVPA and the practice of sports, especially individual sports, and did not smoke. The use of alcohol was reported by a high proportion of EDs users, especially between meals and mainly 2–4 times a month. In comparison with non-users, they showed significantly higher proportions of males (*p* < 0.001), attendants from Northern universities (*p* = 0.004) and life science courses (*p* < 0.001), higher father’s educational level (*p* = 0.003), overweight participants (*p* < 0.001), individuals following particular diet regimens (*p* < 0.001), higher engagement in MVPA (*p* < 0.001) and higher participation in sport (*p* < 0.001) and in team sports (*p* < 0.001), higher amount of smokers (*p* < 0.001) and alcohol users (*p* = 0.005), and higher frequency of alcohol use (*p* = 0.003).

Table 2 reports the main features related to EDs consumption in the sample examined.

**Table 2:** Features of caffeinated ED consumption declared by users (*n* = 329)

Variable	Answer *n* (%)
Frequency of use	
Once a month	136 (41.5)
Once a week	66 (20.1)
Sometime a week	81 (24.7)
Once a day	29 (8.8)
More than once a day	16 (4.9)
Reason for use	
Professional’s indication	140 (46.3)
My own knowledge	65 (21.5)
Relative/friend/teammate’s suggestion	50 (16.6)
Trainer’s suggestion	28 (9.3)
Advertising	19 (6.3)
Aim pursued	
Mental performance	69 (28.0)
Physical performance	64 (26.0)
Taste	45 (18.3)
Improve health	41 (16.6)
Supplementation	21 (8.5)
Physical appearance	4 (1.6)
Recovery	2 (0.8)
Purchasing channel	
Shop/supermarket	251 (80.7)
Specialized website	42 (13.5)
General trade website	10 (3.2)
Sport facility	8 (2.6)
Adverse events	
Gastrointestinal disorders	10 (26.3)
Sleep disorders	8 (21.0)
Irritability	5 (13.2)
Excitement	5 (13.1)
Anxiety	4 (10.5)
Trembling	2 (5.3)
Sweating increase	2 (5.3)
Urination problems	1 (2.6)
Dizziness	1 (2.6)

The most reported frequency of EDs use was once a month. The majority of the consumers declared that they use EDs following a physician/nutritionist’s indication and pursuing an improvement in mental performance, buy them at shops/supermarkets, and reported gastrointestinal disorders as the main adverse event.

Table 3 reports the results of the logistic regression analysis performed considering the use of EDs as the outcome and those variables which appeared to be significantly different between users and non-users.

**Table 3:** Results of the logistic regression performed considering ED consumption as outcome

Variable	Odds ratios (CI95%)
Gender	
Male	*Reference*
Female	0.546 (0.374–0.798)*
Geographical area	
North	*Reference*
Center	0.500 (0.275–0.909)*
South	0.699 (0.470–1.041)
Father’s educational level	
Mandatory	*Reference*
High school	1.110 (0.684–1.802)
Degree	1.330 (0.800–2.212)
BMI	
Underweight	*Reference*
Mormal weight	1.033 (0.464–2.300)
Overweight	1.577 (0.636–3.910)
Obese	0.812 (0.178–3.703)
Diet	
No particular regimen	*Reference*
Mediterranean diet	0.587 (0.362–0.951)*
Other diets	0.977 (0.643–1.485)
Smoke	
No smoker	*Reference*
Smoker	1.712 (1.176–2.492)*
Quitter	0.920 (0.386–2.195)
Playing sport	
No	*Reference*
Yes	0.742 (0.441–1.249)
Sport type	
Individual	*Reference*
Team	1.686 (1.051–2.707)*
Alcohol use	
No	*Reference*
Yes	1.011 (0.622–1.643)

^*^p < 0.05

Being female, attending universities in the Center of Italy and following a Mediterranean diet seems to be negatively related to EDs use, while tobacco smoking and practicing team sport are positively associated with this habit.

## Discussion

This study highlights a slight prevalence of ED consumption among Italian undergraduates (15.2%). In the comparison with non-users, ED users showed a higher proportion of males and a higher father’s educational level, came mainly from Northern universities and degree courses in the life sciences area, and lived in the university area. As for lifestyles, ED users reported higher BMI values, more particular dietary regimens, higher levels of weekly MVPA and participation in sport and in team sports, and a higher proportion of smokers and alcohol drinkers. In the regression analysis gender, diet, geographical area, tobacco smoke and participation in sport were found to be associated with ED consumption.

Although at least weekly frequency of EDs use has been reported by the majority of consumers, the prevalence rate found in our investigation is lower than those (ranging from about 20 to 60%) reported in other studies performed among undergraduates worldwide [23–25] and also in Italy [26–29]. However, it should be noted that these studies show a great heterogeneity in type of products considered, time to which the use was referred, and participants’ characteristics. With specific reference to the previous Italian studies, all of them investigated the use of EDs in students from specific geographical or study areas [26–29].

In our study, undergraduates from 8 regions throughout the Italian territory were involved; since it was reported that energy drinks can be confused with sport drinks, we asked them to refer about the use of caffeinated energy drinks, specifying the difference with sport drinks and suggesting them some examples of commercial products [23, 30]. Therefore, it is possible that these aspects have influenced the prevalence of EDs users in our sample.

The higher use of EDs among males is in line with other studies which examined gender differences [24–26, 29]. In their study, Attila et al. have shown a different prevalence of EDs users between medical students and those from other study areas [23]. Contrarily to them, we found a higher amount of EDs users among life science students. However, this relationship was not confirmed in the regression analysis. In the same study, Attila et al. found a correlation between EDs use and high monthly income [23]. Assuming that monthly income can mirror the parents’ educational level, our high amount of EDs users with graduated fathers is in line with that result. The finding regarding geographical difference is interesting and requires further investigation.

As for lifestyle, EDs users from our sample showed higher proportions of overweight individuals, smokers and alcohol consumers than non-users.

These findings support the notion that EDs consumption is associated with overweight and obesity, mainly due to their content in sugar [31–33]. Interestingly, while the majority of EDs users reported non-common and non-Mediterranean diets, the proportion of those who followed a weight-loss diet was lower than that observed in non-users. The Mediterranean diet was inversely related to the use of EDs. Literature shows that high levels of health literacy and healthy lifestyles are common in individuals who adopt this dietary pattern [30, 31]. It is possible therefore that this diet model can be protective even towards the use of EDs.

Even though in our study a higher proportion of alcohol drinkers was detected among ED users, these habits were not found to be significantly associated in the regression analysis. However, previous studies have indicated that consuming EDs is connected to an excessive consumption of alcohol, even in other population groups [31, 36–38]. Thus, EDs users could be considered a risk group for alcohol abuse and its consequences such as palpitations and other cardiological issues [39]. Among undergraduates, the association between EDs use, smoking and alcohol drinking is common, so as the habit of mixing EDs with alcohol [23–25, 40]. A previously reported cross-sensitization theory could explain how excessive caffeine in EDs could damage the immature reward system in the brain of youths and cause hyper-responsiveness to tobacco and alcohol [41, 42]. Unfortunately, our questionnaire did not investigate the consumption of alcohol mixed with EDs. Further studies should be performed on this specific issue.

As for the association found with MVPA and sport, our results agree with other studies [23, 25] and are in line with the physical performance aim declared by EDs users. In fact, the EDs’ purported benefits of increased energy and enhanced physical performance are commonly advertised, and the ergogenic effect of caffeinated energy drinks is now recognized [43]. In particular, ED caffeine intake may be helpful to support those high-intensity and power-based movements such as running, sprints or jumps typical of team sports, which in our sample were significantly related to the use of EDs [43]. Even though in our sample a little proportion of consumers declared to use EDs following a trainer’s indication and to buy them in sport facilities, it could be opportune to enhance the information about these products in sport and physical activity settings, even to reduce the adverse effects possibly related to EDs consumption [44]. With regard to this, gastrointestinal and sleep disorders were the most frequently reported side effects in our sample.

Some strengths and limitations can be identified for our study. To our knowledge, this is the first study in our country investigating EDs use in a wide student population including different degree courses from Northern, Central, and Southern Italy universities. Furthermore, we asked participants to report their EDs use considering the previous six months. As reported in the literature, this time frame can be appropriate for capturing both chronic and acute use of dietary supplements such as EDs, which can be variable over time [22].

However, being the universities chosen by convenience and not randomly, the sample cannot be considered representative of the whole Italian undergraduate population. Furthermore, the data collected were self-reported and possibly inaccurate.

Finally, the data collection period should be considered when analyzing the results. This study was performed throughout the 2021/2022 academic year, when the COVID-19 pandemic was still ongoing in Italy even though the corresponding control measures were being gradually relaxed. Since the isolation measures related to the pandemic had notable consequences on people’s wellbeing and behaviors [45, 46], it is possible that even EDs consumption changed in the population examined during that emergency situation. In fact, there is some evidence that students increase EDs use during quarantine periods as a way to cope with stress and boredom [47]. However, our study was not aimed at detecting changes that occurred in ED consumption habits during the COVID-19 pandemic. This represents an interesting issue and should be further investigated.

## Conclusions

A low prevalence of EDs users was found in this study. Higher proportions of overweight individuals and alcohol drinkers were found among EDs users than in non-users. EDs consumption seems to be related to male gender, tobacco smoking and engagement in team sport. These findings could encourage figures engaged in education to implement information campaigns aimed at increasing students’ awareness on this issue, in order to prevent or address the excessive use of EDs and the associated unhealthy behaviors.

## Data Availability

The datasets generated during and/or analysed during the current study are available from the corresponding author on reasonable request.
